# Effect of health belief model-based training and social support on the physical activity of overweight middle-aged women: a randomized controlled trial

**DOI:** 10.3389/fpubh.2024.1250152

**Published:** 2024-01-31

**Authors:** Masoumeh Faghih, Mohammad Hossein Kaveh, Mahin Nazari, Khadijeh Khademi, Jafar Hasanzadeh

**Affiliations:** ^1^Department of Health Promotion, School of Health, Shiraz University of Medical Sciences, Shiraz, Iran; ^2^Research Center for Health Sciences, Institute of Health, Department of Health Promotion, School of Health, Shiraz University of Medical Sciences, Shiraz, Iran; ^3^Department of Health Promotion, School of Health, Shiraz University of Medical Sciences, Shiraz, Iran; ^4^Student Research Committee, School of Health, Shiraz University of Medical Sciences, Shiraz, Iran; ^5^Department of Epidemiology, School of Health, Shiraz University of Medical Sciences, Shiraz, Iran

**Keywords:** physical activity, health belief model, social support, overweight, HBM

## Abstract

**Introduction:**

The highest incidence of overweight among adults is found among women, predominantly middle-aged women. While it has been demonstrated that being overweight increases mortality by compromising physical and mental health, it also imposes substantial costs on the healthcare system. Lack of physical activity is a primary contributing factor to becoming overweight. The majority of inactive adults are women, particularly middle-aged women. Consequently, this study investigated the training program for overweight women based on the health belief model (HBM) and social support approach.

**Methods:**

A randomized, controlled trial involving 73 overweight middle-aged women (control group: 37, intervention group: 36) was conducted using simple random sampling. The intervention group participated in six 120-min sessions per week for 6 weeks of a training program based on HBM and social support through physical activity, group discussion, role play, and media. Data were collected using the Physical Activity Questionnaire (IPAQ), Bandura’s Exercise Self-Efficacy Scale (Bandura’s ESE), and a researcher-made questionnaire before and 4 weeks after the training. The collected data were analyzed using descriptive and inferential statistics via SPSS 27 software. *p*-values <0.05 were considered statistically significant.

**Results:**

A training program based on HBM and social support led to improved perceived benefits (*p* < 0.001), cues to action (*p* = 0.03), and self-efficacy (*p* < 0.001) of physical activity; decreased perceived barriers (*p* = 0.001); increased social support (*p* = 0.001); and increased physical activity (*p* < 0.001). In addition, the BMI of the intervention group decreased after the training program (*p* = 0.01).

**Conclusion:**

The findings of the study demonstrate the efficacy of the training program based on HBM and the social support approach in improving social support and physical activity of women. In addition, the study evaluates the long-term outcome in populations with varying social, economic, and cultural standings.

**Clinical Trials Registration:**

https://clinicaltrials.gov/, (IRCT201706236261N17).

## Introduction

The World Health Organization (WHO) reported that more than 1.9 billion adults aged 18 and older were overweight [25 ≤ body mass index (BMI) <30], comprising 39% of the global adult population (1, 2). In both developed and developing countries, the prevalence of overweight women was greater than that of overweight men (2). In Iran, 22.7–38% of adults older than 18 are overweight, with approximately 35–36.82% of women falling into this category. A higher prevalence is observed among middle-aged women (35–65 years) (3–5).

The Centers for Disease Control and Prevention (CDC) stated, “People who are overweight or obese are at a greater risk for many serious diseases and health conditions than those with a healthy weight. These include all causes of mortality, hypertension, dyslipidemia, type 2 diabetes, low quality of life, depression, anxiety, and sleep apnea, among others.” (6). In addition, by 2020, the global healthcare costs associated with overweight and obesity will reach $7.4 billion (7).

Various factors contribute to overweight and obesity, including social–physical environment, genetics, medical history, and behaviors; however, sedentary lifestyles and lack of physical activity are the leading global causes of these conditions (7–9). Globally, 28% of adults and 32% of women aged 18 and older are not physically active enough. In addition, 35% of women in high-income countries and 24% in low-income countries are inactive, and women are less active than men (10). In Iran, 51.3–54.7% of the adult population is inactive, including 57.8–61.9% of women, and the prevalence was highest for middle-aged women (11, 12).

Therefore, as a health strategy, it is necessary to promote and protect the health of overweight women, particularly those in the middle age group, by increasing their physical activity levels (13, 14). Consequently, establishing positive health beliefs through education and training affects physical activity motivation and conduct (15). The health belief model (HBM) applied to various age and cultural groups is the earliest and most applicable model that explains and predicts physical activity based on individual beliefs and is more concerned with prevention (16–18). Four components comprise the original HBM model: perceived benefits, perceived susceptibility, perceived severity, and perceived barriers. Later, self-efficacy and action cues were included in the model. Therefore, the model now includes six key domains (19).

Based on the previous studies, the impact of using HBM constructs on physical activity can be summarized as follows: Perceived susceptibility refers to the belief that inactivity is associated with a higher risk of diseases such as obesity and diabetes, so it is more likely to engage in physical activity as a preventive measure (20); perceived severity relates to the understanding of the consequences of inactivity, ranging from illness to death, so a clear understanding of the seriousness of these consequences motivates lifestyle changes and engaging in physical activity (21); perceived benefits involves the perception of the benefits that can be derived from exercise, whether they are physical, psychological, or social, so it creates a positive mindset and paves the way for action and behavior change (20); perceived barriers are factors or obstacles that may prevent individuals from engaging in physical activity. Recognizing and dealing with these barriers **are crucial for increasing exercise participation (15). Cues to action are prompts or reminders that encourage individuals to engage in physical activity. One important cue is the expectations of family members, which can serve as a strong motivator for individuals to initiate and maintain regular exercise routines (22). Self-efficacy refers to the belief of an individual in their ability to perform physical activities correctly, even under challenging conditions. When individuals have high self-efficacy beliefs, their confidence increases, leading to a higher probability of engaging in physical activity (21).

The two most important components of the model for physical activity are self-efficacy and perceived barriers; both are influenced by social support, which helps to strengthen interventions and the continuation of the behavior (17, 23–27). Four dimensions can be used to define, measure, and present social support: emotional (empathy and love), instrumental (tangible assistance), informational (consulting), and evaluation (constructive feedback) (28). HBM and social support structure can be utilized as a framework for designing and implementing educational interventions to promote self-care behaviors of women (29).

Studies have looked into the impact of HBM-based educational interventions and social support on physical activity (16, 18, 30, 31), but there is a lack of research specifically focusing on the combined effect of these approaches on the physical activity of women. Further research in this area is needed to better understand how to effectively promote physical activity among Iranian women. Therefore, this study aimed to examine the effects of HBM-based training and social support on the physical activity of overweight middle-aged Iranian women.

## Materials and methods

### Ethical considerations

This study was conducted with the clinical trials registration number IRCT201706236261N17 with a registration date of 19/07/2017. Furthermore, all methods were performed per applicable guidelines and regulations. Participants were asked to sign informed consent forms and were provided information about the objectives and procedures of the study.

### Study design and population

This educational randomized controlled trial was conducted in Shiraz, Iran, in 2018–2019. The study population comprised middle-aged women enrolled in the “middle-aged health program” at health centers affiliated with Shiraz University of Medical Sciences. The first important component of HBM for physical activity is self-efficacy, which strengths interventions and sustains the behavior (17, 23–27), so based on the mean ± SD difference of the self-efficacy score in the intervention group (56.14 ± 3.76) from a similar study (14), the sample size was calculated as 40 participants in each group (total 80 women) using the relevant formula (32), a type I error rate of 0.05%, a test power of 95%, and a 20% attrition rate.

Participants were recruited using selected, multistage cluster sampling. In the first phase, we randomly selected one center from two main health centers and four sub-health centers of the city of Shiraz. Two centers were randomly assigned to the intervention and control groups. Random assignment was performed at the cluster level (health centers) to prevent data contamination between the intervention and control groups. Each center was sampled using a standard random technique. As a result, the list of health file numbers of women with a high BMI whom the centers covered served as the sampling basis.

**Figure 1:**
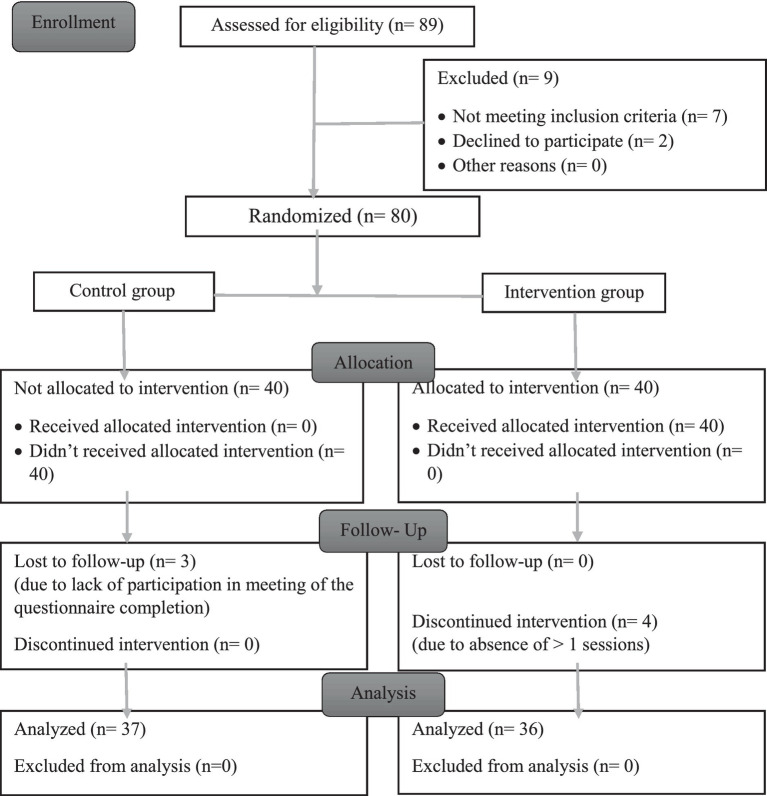
CONSORT diagram of the study.

The sampling interval was determined using a randomization number ranging from 1 to 10. The selected women were evaluated based on the inclusion criteria, and if ineligible, the next woman on the list was chosen. All participants were women aged between 30 and 59, had a BMI between 25 and 29.9 (overweight), had the mental and physical capacity to answer the questions, were not pregnant, and had no physical activity-limiting diseases. Another criterion for inclusion was literacy for reading and writing. Exclusion criteria included missing more than one session, becoming pregnant, developing physical activity limitation conditions, and unwillingness to continue participation. Figure 1 displays the CONSORT (*Consolidated Standards of Reporting Trials*) diagram of the study.

### Data collection

To calculate the BMI ( kgm2 ), height and weight were measured with minimal clothing and bare feet using a digital scale and a wall-mounted meter, respectively. The reliability of a weight scale was determined by measuring one individual five times and calculating the correlation coefficient between the obtained values, which was 0.85.

Participants were required to complete three questionnaires: The Persian version of the International Physical Activity Questionnaire (IPAQ) (33), Bandura’s Exercise Self-Efficacy Scale (Bandura’s ESE) (34), and a researcher-made questionnaire. The reason for selecting questionnaires was their specific ability to measure the variables under consideration, their strong standardization within the Iranian population, and the absence of a similar Persian version.

The IPAQ contained 21 items and was used to measure physical activity levels. In addition, this instrument categorizes many studies that have endorsed the population validity and reliability into three groups: low (not meet criteria for moderate or high level), 600 ≤ moderate<3,000, and high physical activities≥3,000 based on MET (Metabolic equivalent)-min/week scores or the frequency of activities at weekdays and the time spent on each time (35). The intraclass correlation coefficient (ICC) was above 0.70. In addition, the Spearman–Brown correlation coefficient was reported to be 0.9 (33, 35). The original versions of the questionnaire were used for scoring.

The second questionnaire, Bandura’s ESE, was the assessment of the self-efficacy construct. This scale, designed by Bandura (1997), contained 18 items, with a score range of 0 (I cannot do) to 100 (I’m sure I can do). Therefore, individuals with a total score of 0 were not at all confident in their exercise skills, while those with a score of 1800 were the most confident. In previous studies, Cronbach’s alpha was 0.93, and internal consistency was 0.95 (34, 36, 37). The original versions of the questionnaire were used for scoring.

The third was a questionnaire created by a researcher consisting of the following three sections: Part 1 consists of 10 questions regarding age, level of education, marital status, employment status, number of children, presence of chronic diseases, history of tobacco use, history of medication, history of therapeutic diet, and history of sports participation (in every stage of life). Part 2: The HBM constructs (excluding self-efficacy) regarding physical activity. Part 3: Social support for physical activity. The content and construct validity of the researcher-made questionnaire (parts 2 and 3) were evaluated and confirmed by 10 health education specialists on a panel of experts (CVR > 0.89 and CVI > 0.91). Cronbach’s alpha was used to determine the internal consistency of these parts, and values between 0.73 and 0.84 were obtained, indicating the acceptable reliability of the questionnaire. The external reliability of the questionnaire was also evaluated by the test–retest method over a 2-week interval on a sample of 25 middle-aged women (Table 1).

**Table 1:** Researcher-made questionnaire-based HBM constructs in part 2 and social support in part 3.

Part/Constructs	Example of items	Scale	Number of items	Range of scores	Cronbach’s alpha
HBM constructs		5-point Likert scale	47	_	_
Perceived susceptibility	I am more susceptible to illness than others.	1 = strongly disagree, 2 = disagree, 3 = no idea, 4 = agree, 5 = strongly agree	6	6–30	0.90
Perceived severity	I can develop dangerous diseases due to inactivity.		8	8–40	0.90
Perceived benefits	I can wear whatever I like with regular and adequate physical activity.		10	10–50	0.90
Cue to action	When I receive information about being overweight and exercising on TV, I exercise.		14	14–60	0.91
Perceived barriers	Physical activity requires equipment that I do not have.	1 = strongly agree, 2 = agree, 3 = no idea, 4 = disagree, 5 = strongly disagree	9	9–45	0.70
Social support	My family informs me of safe walking areas.	3-point Likert scale 0 = never, 1 = sometimes, 2 = always	10	0–20	0.94

Pre-test and demographic data were collected 1 week prior to the commencement of training sessions during the introduction day. Post-test (post-intervention data) were gathered 4 weeks (1 month) after the conclusion of the intervention, as research suggests that repeating a health behavior requires at least 18 days to become habitual (38).

### Procedure and intervention

**Figure 2:**
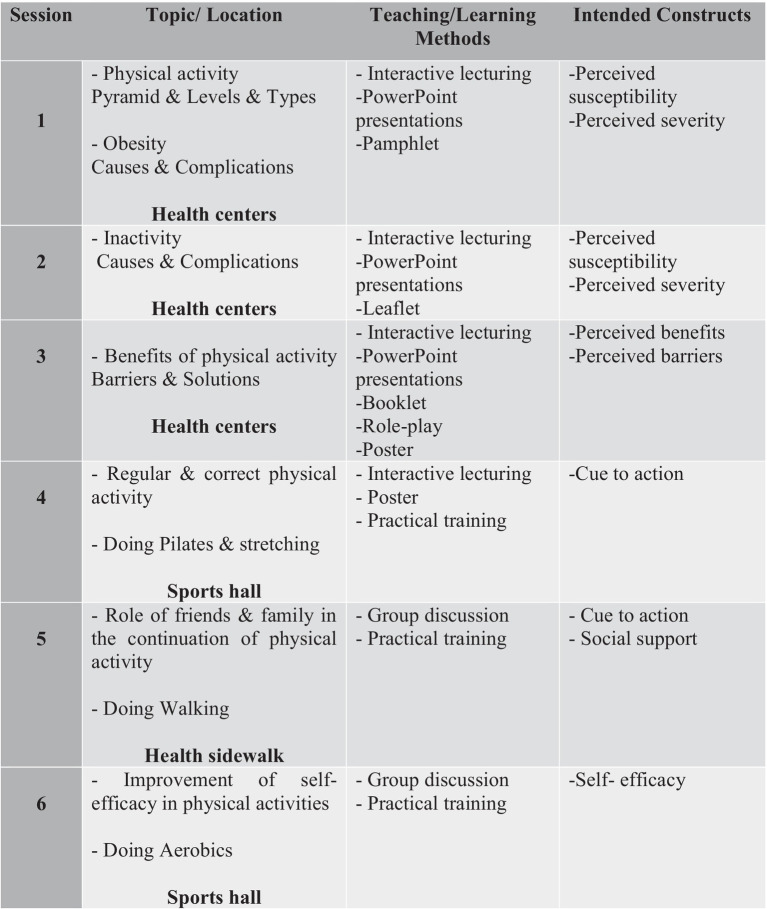
Topics as well as teaching/learning methods and construction focus of each training session.

The intervention group participated in 120-min weekly sessions of physical activity training for 6 consecutive weeks. The number and format of these sessions were determined based on the constructs of the HBM and social support, educational needs identified through previous studies, and participant preferences (39). Beginning with session 4, the first 60 min of each session were devoted to theoretical content education by a researcher with an MSc in health education and promotion. The following 60 min were devoted to practical training and implementation of aerobics, Pilates, stretching, and regular walking with full inhalation and exhalation by an instructor with a master’s degree in physical education approved by the Sports–Cultural–Art Organization of Shiraz Municipality for groups of 10 participants. The content of the session was based on HBM and social support theory concepts. Figure 2 summarizes the topics of each training session, teaching/learning methods, and intended constructs.

Free transportation to and from sports facilities, childcare for mother participants, and educational pamphlets for husbands and families were provided to strengthen social support for the intervention group. In addition, the researcher created a channel on the Telegram® messaging application titled “Health Followers” and sent six motivational messages containing animation images of appropriate physical activity to perform at home to the intervention groups, their families, and their friends. In the end, the top 12 participants in physical activities received prizes.

### Statistical analysis

The collected data were analyzed with descriptive and inferential statistics using SPSS 27 software (IBM Corp., Armonk, NY, United States) (40). Data normality was checked by the Kolmogorov–Smirnov test. In addition, a chi-square test was applied to the demographic data analysis. Moreover, paired and independent *t*-tests were conducted to compare the means of the research construct. The significance level was set at <0.05.

## Results

The descriptive findings of this study revealed statistically significant differences between the control group (*M* = 39.46, SD = 7.86) and the intervention group (*M* = 44.86, SD = 6.71) in terms of mean age (year) [*t* (71) = 2.96, *p* = 0.004]. In addition, there were statistically significant differences in the mean number of children between the control group (*M* = 1.75, SD = 0.64) and the intervention group (*M* = 2.34, SD = 1.02) [*t* (57) = 2.98, *p* = 0.005]. Table 2 summarizes the frequency distribution of other demographic data in the control and intervention groups.

Accordingly, the two study groups had a statistically significant difference in sports activity history [*X*^2^ (1) = 8.504, *p* = 0.004]. Furthermore, there were no significant differences in other characteristics between the two study groups. Most participants held a high school diploma, were married, were homemakers, did not have a history of tobacco use or a therapeutic diet, and did not suffer from chronic diseases.

Except for perceived severity [*t* (71) = 2.09, *p* = 0.02] and self-efficacy [*t* (71) = 1.66, *p* = 0.04], there were no significant differences between the control and intervention groups regarding the mean score of HBM constructs regarding a physical activity before the intervention (Table 2).

**Table 2:** Demographic characteristics of the participants in the study groups.

Characteristics	Control group		Intervention group		Chi-square test
	*n*	%	*n*	%	
Education Below the twelfth grade Diploma Associate degree Bachelor’s degree Master’s degree or higher	4 16 2 13 2	10.8 43.2 5.4 35.1 5.4	6 22 1 6 1	16.7 61 2.8 16.7 2.8	*p* = 0.33
Marital status Married Single Divorce or widowed	35 1 1	94.6 2.7 2.7	31 1 4	86.1 2.8 11.1	*p* = 0.36
Employment status Government employee Homemaker Other	9 26 2	24.3 70.3 5.4	4 28 4	11.1 77.8 11.1	*p* = 0.26
Chronic diseases^a^	10	27.8	15	42.9	*p* = 0.18
History of tobacco use^a^	2	5.6	0	0	*p* = 0.15
History of medication use^a^	12	33.3	12	32.4	*p* = 0.93
History of therapeutic dieting^a^	3	8.1	6	17.1	*p* = 0.24
History of sports activities^a,b^	25	67.6	34	94.4	*p* = 0.004

*N* = 73 (*n* = 37, *n* = 36 for control and intervention groups, respectively). ^a^Reflects the number and percentage of participants answering “yes” to this question. ^b^Reflects “in every stage of life”.

However, 4 weeks after the intervention, a significant difference was observed between the two groups on perceived benefits [*t* (71) = 3.55, *p* < 0.001], perceived barriers [*t* (71) = 3.60, *p* = 0.001], cue to action [*t* (70) = 1.91, *p* = 0.03], and self-efficacy [*t* (71) = 3.45, *p* < 0.001] with effect sizes of very large, large, medium, and small, respectively. In the intervention group, post-test scores on perceived benefits [*t* (33) = −2.82, *p* = 0.02], cue to action [*t* (33) = 2.17, *p* = 0.03], and self-efficacy [*t* (33) = 2.46, *p* = 0.01] differed significantly from pre-test scores; the effect size was medium (Table 3).

**Table 3:** The mean scores of constructs of HBM in the control and intervention groups.

Groups	Before intervention		4 weeks after Intervention		*t* (df)	*p*	Cohen’s *d*
	Mean	SD	Mean	SD			
*Perceived susceptibility*							
Control	24	5.08	24.35	3.32	0.39 (36)	0.69	0.07
Intervention	24.55	3.72	25.65	3.23	1.75 (34)	0.08	0.31
*t* (70)	−0.22		1.35		_	_	_
*p*	0.59		0.09		_		
Cohen’s *d*	1.07		1.29		_		
Perceived severity							
Control	30.13	6.48	31.27	3.48	0.94 (36)	0.35	0.20
Intervention	33.05	4.01	33.66	4.31	0.70 (35)	0.84	0.14
*t* (71)	2.09		1.23		_	_	_
*p*	0.02		0.11		_		
Cohen’s *d*	0.54		0.61		_		
*Perceived benefits*							
Control	43.27	7.09	40.94	6.01	2.44 (36)	0.008	0.35
Intervention	43.77	4.80	46.30	4.47	−2.82 (35)	0.02	0.54
*t* (71)	−0.58		3.55		_	_	_
*p*	0.72		<0.001		_		
Cohen’s *d*	0.08		1.01		_		
*Perceived barriers*							
Control	29.45	5.79	28.94	5.33	0.61 (36)	0.67	0.09
Intervention	31.77	6.89	34.27	7.75	1.67 (35)	0.13	0.33
*t* (71)	1.18		3.60		_	_	_
*p*	0.12		0.001		_		
Cohen’s *d*	0.36		0.80		_		
*Cue to action*							
Control	45.36	7.66	44.72	8.62	−0.35 (35)	0.72	0.07
Intervention	46.33	9.81	50.66	13.51	2.17 (35)	0.03	0.35
t (70)	0.64		1.91		_	_	_
p	0.74		0.03		_		
Cohen’s *d*	0.11		0.52		_		
*Self-efficacy*							
Control	755.94	329.53	745.67	335.87	−0.22 (36)	0.82	0.03
Intervention	924.44	385.25	1061.11	384.78	2.46 (35)	0.01	0.35
*t* (71)	1.66		3.45		_	_	_
*p*	0.04		<0.001		_		
Cohen’s *d*	0.92		1.16		_		

Mean parameter values for each of the analyses are shown for the control group (*n* = 37) and intervention group (*n* = 36) as well as the results of t-tests (assuming unequal variance) comparing the parameter estimates between the two groups.

The findings demonstrated significant differences between the control and intervention groups in the pre-test social support score [*t* (71) = 1.77, *p* = 0.04]; however, the effect size was small. In addition, after the intervention, there was a significant difference between the control and intervention groups in this regard [*t* (71) = 3.10, *p* = 0.001], indicating that the effect size was large. However, in the control and intervention groups, no significant difference was observed in the social support scores at different stages of the study (Table 4).

**Table 4:** The mean scores of social support, BMI, and physical activity in the control and intervention groups.

Groups	Before intervention		4 weeks after intervention		*t* (df)	*p*	Cohen’s *d*
	Mean	SD	Mean	SD			
*Social support*							
Control	7.86	3.37	7.64	3.47	−0.41 (36)	0.67	0.06
Intervention	9.47	3.38	10.88	4.25	1.90 (35)	0.06	0.36
*t* (71)	1.77		3.10		–	–	–
*p*	0.04		0.001		–		
Cohen’s *d*	0.47		0.83		–		
*BMI*							
Control	27.53	1.77	27.41	1.92	−1.34 (36)	0.18	0.06
Intervention	28.15	2.55	27.88	2.62	−2.63 (35)	0.01	0.10
*t* (71)	0.74		0.30		–	–	–
*p*	0.23		0.38		–		
Cohen’s *d*	0.28		0.20		–		
*Physical activity*							
Control	1377.97	1077.18	1990.41	1494.48	2.80 (36)	0.003	0.45
Intervention	1285.83	881.07	4649.28	1670.42	12.58 (35)	<0.001	2.25
*t* (71)	−0.49		3.55		–	–	–
*p*	0.69		<0.001		–		
Cohen’s *d*	0.09		1.67		–		

Mean parameter values for each of the analyses are shown for the control (*n* = 37) and intervention groups (*n* = 36) as well as the results of t-tests (assuming unequal variance) comparing the parameter estimates between the two groups.

The results revealed no significant differences between the control and intervention groups regarding BMI and physical activity score at the pre-test stage. Nonetheless, a significant difference was found between the two groups in terms of physical activity following the intervention [*t* (71) = 3.55, *p* < 0.001]; the effect size was very large. In the intervention group, the mean BMI score at the post-test stage was significantly lower than at the pre-test stage [*t* (33) = −2.63, *p* < 0.01], as was the mean physical activity score [*t* (33) = 12.58, *p* < 0.001]. Compared to physical activity, the effect size for BMI was very small. In the control group, the post-test physical activity mean score was significantly different from the pre-test physical activity mean score [t (34) = 2.80, *p* = 0.003]; the effect size was small (Table 4).

## Discussion

The main purpose of the study was to examine the effect of a training program-based combination of HBM and social support on increasing physical activity levels in a population of overweight Iranian women of middle age.

A training program-based HBM and social support intervention significantly increased the mean scores of perceived benefits, perceived barriers, cue to action, and self-efficacy of physical activity, social support, and physical activity. Similarly, Shao et al. reported significance after an HBM intervention, with an increase in the mean score of HBM and physical activity constructs of asymptomatic hyperuricemia (AHU) patients, whereas social support was not assessed (41). The similarity in the number of training sessions, topic covered, and follow-up time may explain the similarity in the results obtained. It is important to note that positive changes in beliefs, perceptions, and self-efficacy toward physical activity have been found to lead to an increase in engagement in such activities (19). Additionally, a study conducted by Hosseini et al. on the HBM demonstrated a significant increase in the mean score of HBM constructs and physical activity levels among women at risk for hypertension (20). These findings suggest that incorporating the HBM into educational programs can effectively facilitate behavior change and reduce the risk of disease (20, 41).

Khodaveisi et al. conducted a semi-experimental study on HBM among teaching staff at the University of Medical Sciences. They reported a significant increase in the mean score of HBM constructs but no significant change in physical activity level (16). Moreover, the semi-experimental double-blinded study on HBM by Shafieian et al. demonstrates a significant increase in the mean score of HBM constructs of pregnant women but no significant difference in the level of physical activity (22), similar to the semi-experimental study of Jorvand et al. among healthcare workers (18). One potential explanation for the observed differences in physical activity levels could be the limited training hours and longer follow-up periods. It is crucial to adjust training hours based on the specific topic being addressed, the chosen training method, and the expected results (39). By ensuring sufficient training time, researchers can better assess the effectiveness of interventions.

Notably, social support was not investigated in any of the presented studies, even though social support emerged as a significant facilitator of physical activity participation. This support can come from various sources such as family members, friends, and healthcare professionals who provide sincere guidance on exercise performance. Encouraging individuals to seek out supportive relationships and fostering an environment that promotes positive social interactions can enhance engagement in physical activity programs (42).

In the present study, a training program reduced BMI in the intervention group 1 month after the intervention, but there were no significant differences between groups. Similarly, Shao et al. and Rezapour et al. conducted an HBM intervention study that demonstrated a significant increase in physical activity and a decrease in BMI in the intervention group but no significant differences between groups (41, 43). Despite the implementation of numerous weight loss programs, achieving successful weight loss is often challenging. Furthermore, even if individuals do manage to lose weight, they often struggle to maintain their progress in the long term. This is why it is crucial to identify psychological factors that can predict successful weight management and improve the effectiveness of intervention strategies (44).

The clear, theory-based, and practical findings of this study can be implemented as training programs for numerous similar health centers. Another strength of this research is that its findings apply to all women. In addition, a combination of HBM and social support not only focuses on individual factors but also emphasizes social support.

Although the current research has several strengths, this study had several limitations that need to be addressed. First, the post-test period was limited to only 1 month due to previous negative experiences with participant non-cooperation and concerns about attrition rates. In addition, there was a notable disparity in the sports participation history across various stages of the participants’ lives between groups, such as childhood and adolescence, rather than being limited to middle age. This discrepancy could potentially act as a confounding factor. However, the lack of a significant difference in the pre-test physical activity score between groups can help mitigate the impact of this confounder. Additionally, the reliance on self-reported data collection, a common limitation in many studies, raises questions about the accuracy and reliability of the findings. Therefore, it is recommended that further research be conducted to overcome these limitations, particularly in populations with diverse social, economic, and cultural backgrounds. Because various factors, such as environmental, social, biological, cultural, and psychological factors, can have an impact on the physical activity patterns of an individual (20). Furthermore, individuals from different sociocultural backgrounds may hold different beliefs about health. For example, those with higher levels of education often have more comprehensive personal health beliefs (15). The environment also plays a crucial role in shaping physical activity behaviors. Yen et al. expressed that “a supportive environment can promote walking and cycling by providing appropriate infrastructure. A higher level of walkability positively influences people’s willingness to engage in walking and physical activity by reducing negative environmental influences” (45). Additionally, financial constraints often act as a significant economic barrier to participating in physical activity (42).

## Conclusion

In conclusion, a training program based on the health belief model (HBM) and social support intervention has the potential to enhance individuals’ perceived benefits, perceived barriers, cue to action, and self-efficacy toward physical activity. This intervention not only addresses individual beliefs, perceptions, motivations, and self-efficacy but also emphasizes tailored social support to overcome barriers. The findings from this type of intervention can serve as evidence for health policymakers to develop and provide free or paid educational and social facilities for individuals seeking care in adopting healthy behaviors. Additionally, healthcare professionals can utilize this program as an educational tool to promote behavior and lifestyle changes among clients interested in adopting healthier habits. In addition, it is important for future research to explore the potential impact of this training program on physical activity and BMI in women of different age groups, socioeconomic backgrounds, and cultural contexts. This will help to determine the effectiveness of the program across diverse populations and ensure that it can be widely implemented. Furthermore, long-term studies are needed to assess the sustained effects of the program on physical activity and BMI in overweight women. Understanding how these changes evolve over time will provide valuable insights into the lasting benefits of the program and inform strategies for long-term success. Briefly, continued research is essential to fully understand the potential of a training program based on the HBM and social support to improve physical activity and BMI in overweight women. By exploring its impact across different populations and over time, we can maximize its effectiveness and ultimately improve the health outcomes for women worldwide.

## Data availability statement

The raw data supporting the conclusions of this article will be made available by the authors, without undue reservation.

## Ethics statement

The studies involving humans were approved by shiraz university of medical sciences. The studies were conducted in accordance with the local legislation and institutional requirements. The participants provided their written informed consent to participate in this study.

## Author contributions

MF collected the data and drafted the original manuscript. MN was the advisor and revised the manuscript. KK re-analyzed the data and edited and reviewed the manuscript. MK supervised and revised the manuscript. JH analyzed the data. All authors contributed to the article and approved the submitted version.
